# Sars-Cov2 Not Detected in a Pediatric Population With Acute Respiratory Infection in Primary Care in Central and Southern Italy From November 2019 to Early March 2020

**DOI:** 10.3389/fped.2021.620598

**Published:** 2021-05-11

**Authors:** Caterina Rizzo, Daniela Loconsole, Elisabetta Pandolfi, Marta Luisa Ciofi Degli Atti, Jojanneke van Summeren, John Paget, Luisa Russo, Ilaria Campagna, Ileana Croci, Francesco Gesualdo, Carlo Concato, Giulia Linardos, Veronica Bartolucci, Sara Ciampini, Andrea Onetti Muda, Massimiliano Raponi, Maria Chironna

**Affiliations:** ^1^Clinical Pathways and Epidemiology, Bambino Gesù Children's Hospital, IRCCS, Rome, Italy; ^2^Department of Biomedical Science and Human Oncology, University of Bari, Bari, Italy; ^3^Multifactorial Disease and Complex Disease Area, Bambino Gesù Children Hospital, Rome, Italy; ^4^Nivel, Netherlands Institute for Health Services Research, Utrecht, Netherlands; ^5^Virology Unit, Laboratory Department, Bambino Gesù Children's Hospital, Rome, Italy; ^6^Public Health Service, Local Health Authority Rome 1, Rome, Italy; ^7^Department of Laboratories, Bambino Gesù Children's Hospital, Rome, Italy; ^8^Medical Direction, Bambino Gesù Children's Hospital IRCCS, Rome, Italy

**Keywords:** SARS-CoV2, ARI, RSV, pediatric cases, epidemiology, primary care

## Abstract

**Background:** In December 2019, a novel coronavirus named SARS-CoV-2 started circulating in China and this led to a major epidemic in Northern Italy between February and May 2020. Young children (aged <5 years) seem to be less affected by this coronavirus disease (COVID-19) compared to adults, although there is very little information on the circulation of this new virus among children in Italy. We retrospectively tested nasopharyngeal swabs for SARS-CoV-2 in samples collected in young children between November, 2019 and March, 2020 in the context of the RSV ComNet study.

**Methods:** Two networks of primary care pediatricians in Lazio (Central Italy) and Puglia (Southern Italy) collected nasopharyngeal swabs from children, aged <5 years, presenting with symptoms for an acute respiratory infection (ARI). The RSV ComNet study is a multicenter study implemented to estimate the burden of RSV in young children (aged <5 years) in the community. Swabs were sent to a central reference laboratory and tested for 14 respiratory viruses through RT-PCR. All collected samples were retrospectively tested for SARS-CoV-2 using RT-PCR (Istituto Superiore di Sanità protocol).

**Results:** A total of 293 children with ARI were identified in the two participating networks. The highest number of cases were recruited in weeks 51/2019 and 3/2020. The majority of patients (57%) came from the Lazio region. All of the 293 samples tested negative for SARS-Cov2. Rhinovirus was the most frequently detected virus (44%), followed by RSV (41%) and influenza viruses (14%).

**Conclusions:** Our study shows that in Lazio (a region of intermediate SARS-COV-2 incidence) and Puglia (a region of low incidence), the SARS-Cov2 virus did not circulate in a sample of ARI pediatric cases consulting primary care pediatricians between November 2019 and March 2020.

## Introduction

In December 2019, a novel coronavirus named severe acute respiratory syndrome coronavirus 2 (SARS-CoV-2), causing COVID-19 (coronavirus disease 2019), started circulating in China and subsequently spread in many countries (1). On the 11th of March, 2020, COVID-19 was declared a pandemic by WHO (2) and on the 30th of January, 2020, Italy reported the first 2 cases of SARS-CoV-2 infection (3–5), both with a history of travel to Wuhan, China (5). The first Italian patient with COVID-19 was diagnosed on February 21, 2020, a 38-year-old man hospitalized at Codogno Hospital, Lodi, in northern Italy, with no travel history to areas where the virus was already isolated or link to a probable or confirmed COVID-19 case (6). On the same day, another outbreak of COVID-19 was reported in Vò Euganeo (Padua) in the Veneto region, with the first death reported in a 78-year-old man hospitalized in Padua (6). Before this date, in Italy only three cases of SARS-CoV2 infection were identified, all with travel history to Wuhan. Subsequently, extensive and ongoing transmission was identified in several municipalities of the Lombardy region (7). Thereafter, case counts, and death tolls rapidly increased, with the majority of cases in northern Italy with few cases reported in the rest of the country. The Italian government recommended strict physical distancing measures closing 10 municipalities in the Lodi Province (Lombardy) and one in the Padua province (Veneto) on the 23rd of February 2020. This culminated in a national lock-down on the 11th of March 2020 (8, 9) that resulted in a reduction of the incidence of the COVID19 pandemic (10). However, as already reported by some countries, measures implemented during the lockdown results in a substantial drop in the number of children completing their vaccination schedule, which could lead to possible further outbreaks of vaccine-preventable diseases (11–14).

Some studies conducted suggest that SARS-CoV-2 might entered Italy between December and early February 2020 a scenario that is compatible with observations in other European (15–18). Very little information is available in the literature on the circulation of SARS-Cov2 in the pediatric population. Recently, one study conducted in Italy identified in sera collected from September 2019 to February 2020 seropositive adults (14.2% in September and 16.3% in October) with IgG or IgM antibodies, or both (19). A recent study demonstrate the presence of a confirmed SARS-CoV2 child ~3 months before the first identified coronavirus disease case in Italy (20). However, children seem to be less affected by COVID-19 than adults are and a large cohort study conducted in Italy in a network of ED showed that hospitalized children may have rare but serious and life-threatening presentations (21). In Europe, as of 13 May 2020, only 0.7% of the 576 024 laboratory-confirmed cases reported to the ECDC was <4 year of age (22). The objective of our study was to characterize the circulation of SARS-CoV-2 among Italian children at the beginning of the COVID-19 epidemic in Italy. To meet this objective, we retrospectively looked for SARS-CoV-2 in nasopharyngeal aspirate samples collected among children aged <5 years, presenting to their primary care pediatricians with symptoms of an acute respiratory infection (ARI). The samples had been collected between November, 2019 and March, 2020 in the context of a study on the epidemiology of the Respiratory Syncytial Virus (RSV).

## Methods

### Study Population

The organization of pediatric primary care in Italy is one in which young children (<6 years old) are generally cared for by a pediatrician and school-age children (6–14 years old) have the option to see either a pediatrician or a general practitioner. We carried out our study using two networks of primary care pediatricians (PCP) - one in the Lazio Region (Central Italy) and one in the Puglia Region (Southern Italy). The participating pediatricians collected the nasopharyngeal swabs from children, younger than 5 years, presenting at their office with ARI symptoms, between week 47/2019 (mid-November 2019) and week 11/2020 (early March).

### ARI Case Definition

The ARI case definition was based on the WHO definition for community-based RSV surveillance (23) and included the following criteria:

1. Acute – defined as a sudden onset of symptoms;
2. Respiratory infection – defined as having at least one of the following: shortness of breath, cough, sore throat, coryza;
3. Clinician's judgement that the illness is due to an infection.

Note: Point (3) was added to the definition by the RSV ComNet research team.

### Study Design and Procedure

The study was conducted in the framework of a multicenter study to estimate the burden of RSV in children <5 years of age in the community (the RSV-ComNet, see: https://nivel.nl/en/RSVComNet). In this study, swabs were sent to a regional laboratory and tested for 16 respiratory viruses (including RSV A and B, influenza virus A and B, human coronavirus OC43, 229E, NL-63 and HUK1, adenovirus, hRV, parainfluenza virus 1-2-3-4, human metapneumovirus-hMPV and human bocavirus-hBoV) through RT-PCR. Informed consent was obtained from parents of enrolled children. At enrollment, PCPs collected preliminary information for each of the recruited patients, including demographic data (birth date, sex, date of swabbing), date of onset of clinical symptoms, and clinical picture at enrollment. Descriptive statistics were used to describe the patient demographics, and clinical symptoms. All data were analyzed using STATA 13®.

### Laboratory Analysis

After being collected and analyzed, all samples were frozen at −80°C in a Thermo ScientificTM −86°C freezer and stored in case of need for further analysis. In May-June 2020, all previously collected samples were tested for SARS-CoV-2, independently from the previous test results. Nucleic acid was extracted using the STARMag Universal Cartridge kit (Seegene) on the automated Nimbus IV platform. SARS-CoV-2 RT-PCR was performed on CFX96 (Bio Rad Laboratories) with AllplexTM 2019 n-CoV Assay according to the Istituto Superiore di Sanità protocol (14), with the targets being the E gene, encoding the envelope protein of Sarbecovirus, RdRp (RNA-dependent RNA polymerase) and N (nucleocapside) genes specific for CoV-2019.

### Ethical Approval

The Medical Ethical Committee of OPBG Medical Center (Italy) approved the original study (prot. 1936_OPBG_2019), and subsequently waived the study for the additional SARS-CoV-2 testing on the 12th May 2020 (prot. N 525).

## Results

We recruited ARI cases from two municipalities located in two Italian regions: Rome in the Lazio region and Bari in the Puglia region. The population of the Roma municipality accounts for 7.2% of the Italian population, and the population of Bari municipality accounts for 2.1% of the Italian population. The total population of children younger than 5 years of age in the two participating municipalities accounted for 5% of the National population aged 0–5 years. The study involved 12 pediatricians from the Lazio region and 12 pediatricians in the Puglia region.

**Figure 1:**
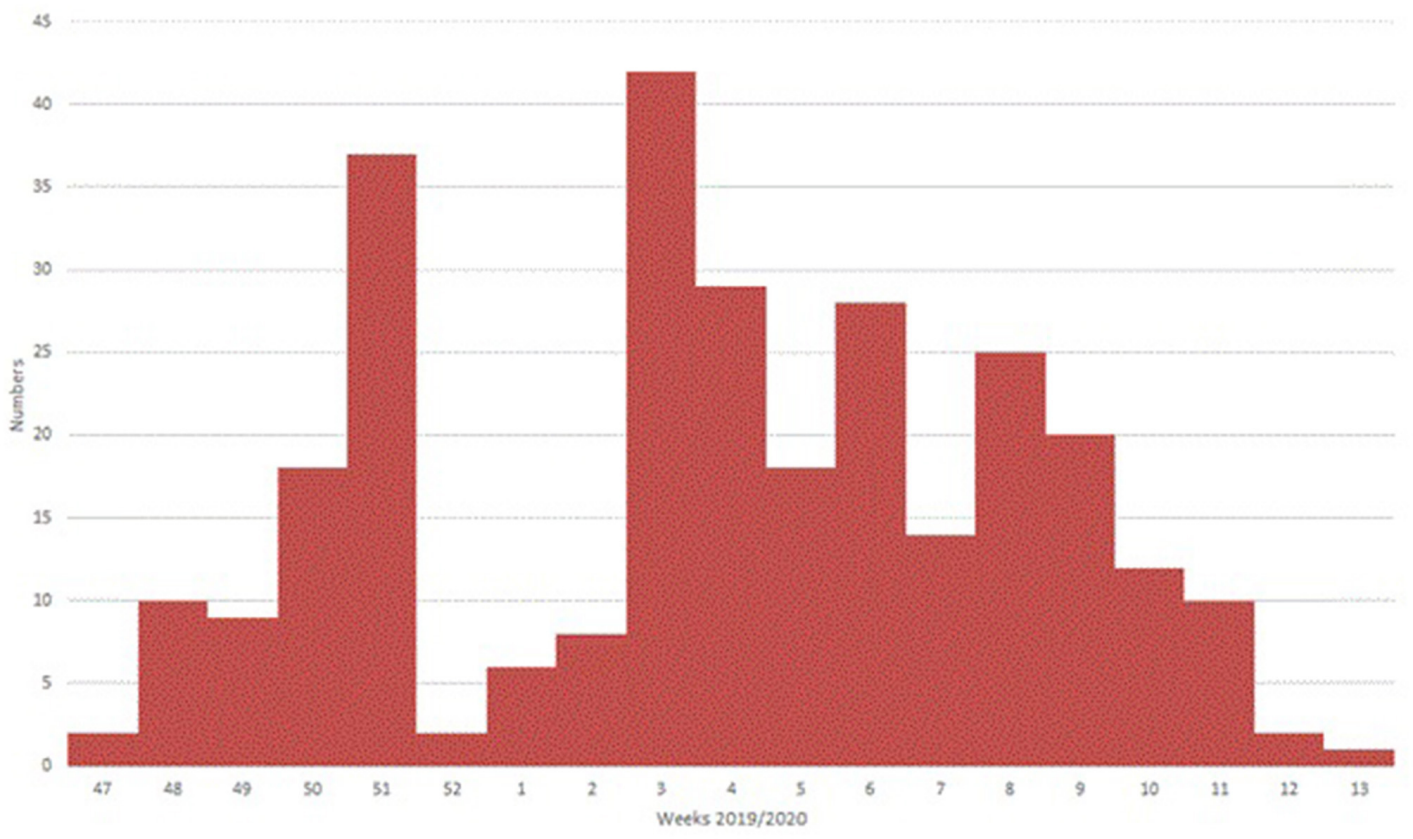
Distribution of total swabs collected in Italy, week 47-2019 to 13-2020.

A total of 293 patients with ARI symptoms presenting to their primary care pediatricians were recruited in the two participating networks (24 participating pediatricians). The highest number of cases were enrolled in week 51/2019 and 3/2020 (Figure 1). The majority of ARI patients (168; 57%) came from the Lazio region, and 125 (43%) were from the Puglia region. Table 1 presents a description of demographic and clinical characteristics of the enrolled patients. Most patients 130 (44%) were younger than 1 year of age, 70 (24%) belonged to the 12–24 months age group and 93 (32.7%) were 24–36 months old. The median number of days between disease onset and swabbing was 2 (IQR 1-3.5). Cough was the most frequently reported symptom (98%), followed by coryza (86%) and shortness of breath (62%). Very few cases were affected by underlying conditions (Table 1). Eighteen cases presented to the ED after the pediatricians visit and seven were hospitalized. All samples tested negative for SARS-CoV-2. Rhinovirus was the most frequently detected virus (44%) followed by RSV (41%) and influenza viruses (14%) (Table 2). A high number of coinfections was observed (215, corresponding to 73.4% of all enrolled patients). Most frequently isolated coinfections were Rhinovirus and Bocavirus (*N* = 8), Rhinovirus + Adenovirus (*N* = 7) and Rhinovirus + Coronavirus OC43 (*N* = 6).

**Table 1:** Description of ARI cases characteristics.

**Characteristic**	**Total (*****n*** **=** **293)**		**Lazio (*****n*** **=** **168)**		**Puglia (*****n*** **=** **125)**	
	***n***	**%**	***n***	**%**	***n***	**%**
**Region**						
Lazio	168	57.3				
Puglia	125	42.7				
**Sex** **=** **male**	159	54.3	87	51.8	72	57.6
**Age group (months)**						
0–12	130	44.4	69	41.1	61	48.8
13–24	70	23.9	48	28.6	22	17.6
25–60	93	31.7	51	30.4	42	33.6
**Pre-maturity**	10	3.5	5	3.1	5	4.0
**Presence of chronic condition**						
Respiratory disease	4	1.4	1	0.6	3	2.4
Malnutrition	1	0.4	1	0.6	-	-
Immunocompromised	0	-	-	-	-	-
Others	4	1.4	1	0.6	3	2.4
**Symptoms**						
Shortness of breath	177	61.7	91	56.2	86	68.8
Cough	286	97.6	162	96.4	124	99.2
Sore throat	122	41.8	88	52.7	34	27.2
Coryza	251	86.3	153	92.2	98	78.4

**Table 2:** Distribution of ARI cases by type of virus and by age group.

			**Age group (months)**						
	**Total**		**0–12**		**13–24**		**25–60**		
	***n***	**%**	***n***	**%**	***n***	**%**	***n***	**%**	***p***
Rhinovirus	128	43.7	60	46.2	32	45.7	36	38.7	*0.848*
RSV	119	40.6	53	40.8	26	37.1	40	43	*0.751*
Adenovirus	30	10.2	6	4.6	11	15.7	13	14	*0.052*
Bocavirus	24	8.2	6	4.6	10	14.3	8	8.6	*0.172*
Coronavirus OC43	21	7.2	7	5.4	6	8.6	8	8.6	*0.786*
Enterovirus	20	6.8	7	5.4	7	10	6	6.5	*0.736*
FLU A(H3N2)	20	6.8	8	6.2	5	7.1	7	7.5	*0.955*
Metapneumovirus	19	6.5	11	8.5	3	4.3	5	5.4	*0.751*
FLU A(H1N1)	11	3.8	4	3.1	4	5.7	3	3.2	*0.849*
FLU B	9	3.1	2	1.5	2	2.9	5	5.4	*0.499*
Parainfluenza 4	6	2	2	1.5	4	5.7	0	-	*0.131*
Coronavirus NL63	6	2	3	2.3	0	-	3	3.2	*0.613*
Enterovirus (HEV)-68	5	1.7	1	0.8	3	4.3	1	1.1	*0.397*
Parainfluenza 1	2	0.7	0	-	1	1.4	1	1.1	*0.711*
Parainfluenza 3	2	0.7	2	1.5	0	-	0	-	*0.579*
SARS-Cov2	0	-	0	-	0	-	0	-	*-*

## Discussion

We show that no SARS-Cov2 infections were detected in nasopharyngeal swabs in a sample of 293 children with ARI symptoms aged 0–5 years, enrolled between November 2019 and early March 2020 in the Italian regions of Lazio and Puglia. COVID-19 caused by SARS-CoV-2 was first reported in December 2019, in the city of Wuhan in the province of Hubei, China, in people who had visited a seafood market (24). By the time the real potential of its pathogenicity was realized, it had spread to many regions of China, other Asian countries, European countries, the United States etc. and by April 20, 2020, it had spread to 185 countries all over the world (25). By February 21, 2020, there were 47 confirmed cases of COVID-19 in the European Region i.e., France (12 positive cases), Germany (16 cases), Belgium (1 case), Finland (1 case), Italy (3 cases), Spain (2 cases), Russia (2 cases), Sweden (1 case) and UK (9 cases). By March 6, 2020, there were 5,544 COVID-19 cases and 159 deaths in the EU and the UK (17 cases) (3). Of all the European countries, Italy was the worst affected in the initial stage. As of late March 2020, all Italian regions and Autonomous Provinces had reported at least one locally acquired case of COVID-19. In particular, there were high incidence regions with sustained local transmission (mainly in the north), low incidence regions (like Puglia) with limited but growing numbers of locally acquired cases of infection and regions with intermediate incidence (like Lazio) (26). Some studies have speculated that SARS-CoV-2 may have been circulating before the first recorded local cases in several countries (15–17). The majority of these studies were phylogenetic, serological or retrospective analyses of mainly adults cases (15, 27–31). While some were the result of modeling studies speculating that in some parts of the country, such as in central (Lazio) and southern regions (Puglia), transmission in adults was largely undetected until the first days of March (21). Nevertheless, few information on the circulation of SARS-CoV2 in the pediatric population in Italy in the community is available, to date (21).

Our study could have some potential limitations. Firstly, it has been argued that nasopharyngeal swab is not the gold standard for diagnosis of SARS-CoV2 in children (32, 33). In our study we swabbed only symptomatic ARI children within 2 days (IQR 1-3.5), on average, from symptoms onset, and, therefore, this could have increased nasopharyngeal swab sensitivity. Another possible limitation is due to the fact that is difficult to generalize our results to the wider population, as we only focused on the pediatric population. However, our study was concentrated only on ARI symptomatic cases presenting to their pediatricians in primary care.

We found a very high proportion of coinfections in our sample with most frequently isolated coinfections being Rhinovirus + Bocavirus, followed by Rhinovirus + Adenovirus and Rhinovirus + Coronavirus OC43. As reported in a recent meta-analysis (34) we observed that RSV and Adenovirus were mostly detected as single infections. This proportion is higher to what observed in previous studies and is probably due to the fact that the number of co-viruses tested in Italy was larger in comparison to the Netherlands, but also larger compared to other published studies (35).

Our study suggests that, based on a sample of ARI pediatric cases consulting primary care pediatricians in low and intermediate incidence areas of Italy from November 2019 to March 2020, the SARS-Cov2 virus did not circulate in children aged < then 5 years. These findings are consistent with the epidemiological situation of COVID-19 in Italy, as the increase in the incidence was observed later in the regions where this study was conducted compared to the northern regions where the first Italian cases occurred and which had the greatest impact (6).

## Data Availability Statement

The raw data supporting the conclusions of this article will be made available by the authors, without undue reservation.

## Ethics Statement

The studies involving human participants were reviewed and approved by The Medical Ethical Committee of OPBG Medical Center (Italy) approved the original study (prot. 1936_OPBG_2019), and subsequently waived the study for the additional SARS-CoV-2 testing on the 12th May 2020 (prot. N 525). Written informed consent to participate in this study was provided by the participants' legal guardian/next of kin.

## Author Contributions

CR conceived the study, coordinated the study, and wrote the article. EP wrote the article and contributed to the interpretation of results. ICr performed the statistical analysis. DL, LR, ICa, FG, CC, GL, VB, and SC coordinated the study at the local level and revised the draft article. JP and JS supported the coordination of the study at the international level and revised the draft article. MLC, AM, MR, and MC revised the draft article and contributed to the interpretation of results. All authors contributed to the article and approved the submitted version.

## Conflict of Interest

The authors declare that the research was conducted in the absence of any commercial or financial relationships that could be construed as a potential conflict of interest.
